# Endothelial cell-cell adhesion and signaling

**DOI:** 10.1016/j.yexcr.2017.06.003

**Published:** 2017-09-01

**Authors:** Camilla Cerutti, Anne J. Ridley

**Affiliations:** Randall Division of Cell and Molecular Biophysics, King's College London, New Hunt's House, Guy's Campus, London SE1 1UL, UK

**Keywords:** Endothelial cells, Adherens junctions, Tight junctions, Rho, GTPases, Rap GTPases, Actin cytoskeleton, Cell signaling

## Abstract

Endothelial cells line blood vessels and provide a dynamic interface between the blood and tissues. They remodel to allow leukocytes, fluid and small molecules to enter tissues during inflammation and infections. Here we compare the signaling networks that contribute to endothelial permeability and leukocyte transendothelial migration, focusing particularly on signals mediated by small GTPases that regulate cell adhesion and the actin cytoskeleton. Rho and Rap GTPase signaling is important for both processes, but they differ in that signals are activated locally under leukocytes, whereas endothelial permeability is a wider event that affects the whole cell. Some molecules play a unique role in one of the two processes, and could therefore be targeted to selectively alter either endothelial permeability or leukocyte transendothelial migration.

## 1 Introduction

**Fig. 1:**
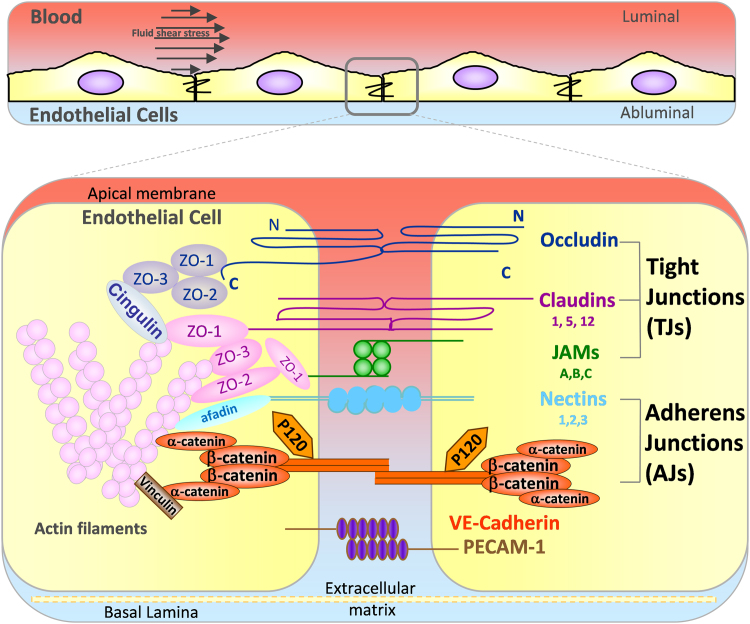
**The main transmembrane proteins in endothelial cell-cell junctions.** Endothelial cells line the blood stream and are constantly exposed to fluid shear stress (top panel). The bottom panel shows the main transmembrane proteins in endothelial cell-cell junctions (right). They are associated with tight junctions or adherens junctions as indicated, with the exception of PECAM-1, which is not associated with either type of junction. Three different claudin, JAM and nectin genes are reported to be expressed in endothelial cells (numbers/letters indicated under protein name). Intracellular proteins link transmembrane proteins to the actin cytoskeleton. There are additional junctional molecules, such as CD99 and ESAM, which are omitted for clarity.

**Fig. 2:**
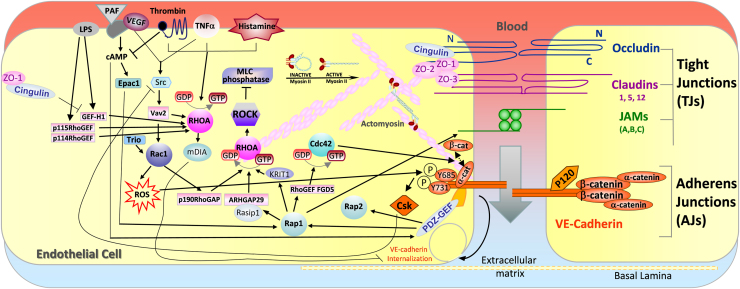
**Signaling molecules that regulate vascular permeability.** The main junctional proteins that regulate vascular permeability are shown on the right cell. The left cell shows examples of receptors that increase vascular permeability, and the intracellular signaling molecules that contribute to this response, focusing on those regulated by Rho and Rap GTPases. ROS, reactive oxygen species.

Endothelial cells (ECs) form a semi-permeable barrier to separate the blood stream from the underlying organs and tissues and control the transport of fluids, solutes and cells across blood vessel walls. The barrier is mediated by endothelial cell-cell adhesions including tight junctions (TJ), adherens junctions (AJ) and a variety of other adhesion molecules including PECAM-1 and nectins, which are connected to the actin cytoskeleton via different adaptor molecules (Fig. 1). The architecture and the composition of cell-cell junctions varies between vascular beds depending on the organ-specific requirements [1], [2]. In addition to maintaining adhesion between ECs, cell-cell junctions control vascular permeability and leukocyte migration via a complex balance between multiple signaling molecules (Fig. 1, Fig. 2) [3], [4].

TJs regulate the diffusion of ions and polar solutes and block penetration of large macromolecules across ECs. TJs are formed by the homophilic cell-cell adhesion molecules occludin, claudins and junctional adhesion molecules (JAMs) (Fig. 1) [5], [6]. Claudins are the principal barrier-forming proteins, in particular claudin-5 is critical for endothelial permeability in vivo and in vitro [7]. Occludin and claudins are linked to zonula occludens (ZO)-1, ZO-2, ZO-3, cingulin and other protein complexes, which mediate the interaction between the adhesion molecules and actin filaments [5]. The JAM family is composed of three closely related proteins JAM-A, -B and -C, and by the coxsackie and adenovirus receptor (CAR). The JAMs mediate endothelial cell-cell interaction and regulate leukocyte transendothelial migration (TEM) [8]. Both JAMs and CAR regulate permeability by supporting TJ function and assembly [9].

VE-cadherin is the key transmembrane component of endothelial AJs and is expressed only in ECs [10]. VE-cadherin together with PECAM-1 initiates and maintains endothelial cell-cell contact, holding the ECs together to give mechanical support to the endothelium and provide endothelial junction stability [11], [12]. AJs can regulate expression of TJ components and TJ organization follows AJ formation [11], [13]. In addition, AJs and TJs are interconnected [14], for example ZO proteins crosstalk with AJs [15]. VE-cadherin is linked indirectly via its cytoplasmic tail to actin filaments by a complex of proteins including α- and β-catenins, plakoglobin (γ-catenin), p120-catenin, vinculin and α-actinin (Fig. 1) [16], which are vital for junctional stability and also for the dynamic opening and closing of junctions [17].

Here we discuss the roles of endothelial cell-cell junctions in signaling leading to changes to endothelial permeability and during leukocyte transendothelial migration (TEM), with a particular focus on small GTPases.

## 2 Regulation of small GTPases

**Fig. 3:**
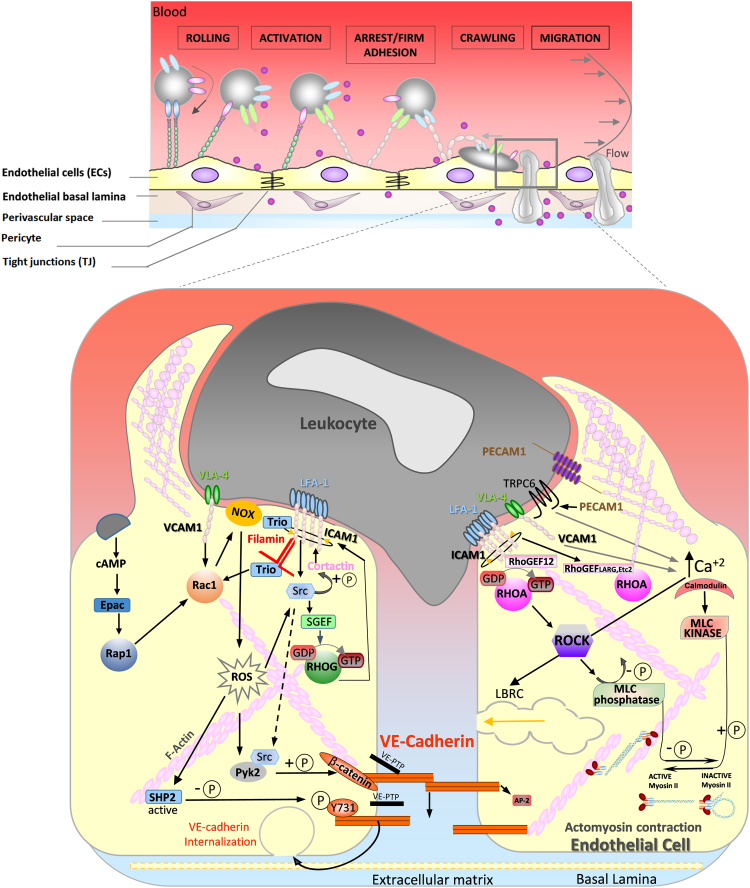
**Signaling molecules that regulate leukocyte transendothelial migration.** Leukocyte attachment to and transmigration across endothelial cells is a multistep process, involving a variety of different adhesion molecules on leukocytes and endothelial cells (top panel). During transendothelial migration, endothelial adhesion molecules ICAM-1, VCAM-1 and PECAM-1 interact with leukocytes and transmit signals into the endothelial cell (bottom panel), which promote the transmigration process. The left cell shows signals involving RhoG, Rac1 and Rap1 GTPases; the right cell shows signals involving RhoA. LBRC, lateral border recycling compartment; ROS, reactive oxygen species.

Several members of the Ras superfamily of small GTPases contribute to endothelial cell-cell adhesion and hence regulate endothelial permeability and/or leukocyte TEM. These include Rap1, Rap2 and several Rho family GTPases (Fig. 2, Fig. 3). Most small GTPases cycle between an inactive GDP-bound conformation and an active GTP-bound conformation. This cycling is regulated by guanine nucleotide exchange factors (GEFs), which catalyse the exchange of GDP for GTP, thereby activating the proteins, and by GTPase-activating proteins (GAPs), which stimulate GTP hydrolysis and inactivate the proteins. The interaction between small GTPases and their downstream effectors often requires their localisation to membranes, which is mediated by post-translational modification by lipids (prenylation and/or palmitoylation) [18], [19]. For example, geranylgeranylation of Rho GTPases is required for thrombin-induced permeability in ECs [20]. Some small GTPases are inhibited by binding to proteins that extract them from membranes, including guanine nucleotide dissociation inhibitors (GDIs) and 14-3-3 proteins [21], [22]. Small GTPases are rapidly activated by cell-surface receptors, and a single GTPase can interact with multiple downstream targets to induce a range of cellular responses in a spatio-temporal fashion, depending on the stimulus and cell type [19].

## 3 Signaling to endothelial junctions in vascular permeability

Endothelial cell-cell junctions remodel dynamically in response to multiple extracellular stimuli, which transiently increase or decrease endothelial permeability and thereby regulate the entry of polar solutes and macromolecules into the tissues from the blood stream (Fig. 2) [4]. For example, pro-inflammatory stimuli such as thrombin, histamine and TNFα increase permeability, whereas anti-inflammatory mediators including sphingosine 1-phosphate (S1P) and angiopoietin-1 decrease permeability [23]. Increased vascular barrier leakage is associated with disruption of endothelial junctions, in particular AJs [10], and occurs in diseases such as cancer, atherosclerosis, diabetes, hypertension, inflammation, and ischemia [24], [25]. In addition, cerebral cavernous malformations (CCMs) are inherited or sporadic diseases that are associated with disorganization of venous brain microvessel junctions and increased permeability [26].

### 3.1 Signals that increase endothelial permeability

Pro-inflammatory stimuli increase permeability through a combination of changes to AJ, TJ and the associated actin cytoskeleton (Fig. 2). AJ composition and integrity is regulated by changes in phosphorylation of junctional proteins. For example, thrombin activates the serine/threonine kinase protein kinase C (PKC)α, which induces VE-cadherin complex disassembly by phosphorylating p120ctn [27]. In VEGF-stimulated ECs, Src mediates Rac1 activation, leading to VE-cadherin phosphorylation via the serine/threonine kinase PAK, and thereby increasing endothelial permeability [28]. LPS activates the Rab family GTPase Rab5, which mediates VE-cadherin internalization resulting in increased permeability [29].

Tyrosine phosphorylation of AJ components is also associated with increased permeability. In unstimulated ECs, receptor and cytoplasmic protein tyrosine phosphatases (PTPs) localize to endothelial AJs and de-phosphorylate AJ components to reduce permeability [17], [30], [31]. For example, the tyrosine phosphatase SHP2 associates with β-catenin in VE-cadherin/p120/plakoglobin complexes [32]. Depletion of SHP2 enhances phosphorylation of AJ complex components leading to increased permeability in response to thrombin [32], [33]. TNFα increases tyrosine phosphorylation of VE-cadherin to enhance vascular permeability [34], [35]. VE-cadherin Tyr685 phosphorylation is required for VEGF- and histamine-induced permeability in vivo [36]. Furthermore, phosphorylation of VE-cadherin on Tyr658 and Tyr685 was reported to be required for increased permeability and endocytosis of VE-cadherin in response to bradykinin and histamine [37].

Endothelial cell-cell contacts are linked to the actin cytoskeleton, which is central to junction stability and contributes to changes in vascular permeability [38]. Permeability-inducing factors destabilize cell-cell junctions by changing actin bundle orientation, which switch from being parallel to junctions to being perpendicular. Perpendicular actin filaments, including stress fibers, impose mechanical force on junctions, and disrupt their integrity [39], [40], [41]. RhoA induces stress fibers and increases contractility leading to higher vascular permeability in response to a variety of extracellular stimuli including thrombin and histamine (Fig. 2). RhoA activates the serine/threonine kinase ROCK, which phosphorylates the MYPT1 subunit of the trimeric myosin light chain (MLC) phosphatase, thereby inhibiting its enzymatic activity [42], [43]. Subsequent increased MLC phosphorylation leads to increased actomyosin contractility. RhoA is required for increased permeability in response to several stimuli including thrombin and TNFα [44]. Interestingly, RhoB and RhoC are also activated by thrombin [45], and RhoB promotes sustained thrombin-induced contraction [46]. By contrast, RhoA activation by angiopoietin-1 leads to cell-cell junction stabilization and inhibits VEGF-induced permeability, and this response is dependent on the Rho-activated formin mDia1 but not ROCK [47]. This suggests that the effect of RhoA on junctions depends on whether it activates ROCK, promoting actomyosin contractility, or on mDia1, which stimulates actin polymerization [48].

Increased permeability often reflects loss of TJ proteins from junctions, which is linked to enhanced RhoA/ROCK signaling. For example, ROCK induces phosphorylation of claudin-5 and occludin [49]. TNFα increases permeability by inducing a decrease in claudin-5 at junctions via ROCK1 and ROCK2 [50]. LPS induces loss of TJ proteins and increased endothelial permeability via activation of RhoA by p115RhoGEF [51]. In the TJ complex, ZO-1 is bound to cingulin which is linked to the actin cytoskeleton (Fig. 1). Cingulin promotes endothelial barrier function in vitro and in vivo*,* in part by interacting with and inhibiting the RhoA GEF-H1 [52], [53], whereas the cingulin-like protein JACOP associates with p114RhoGEF to maintain endothelial TJs and AJs, presumably by stimulating RhoA activity at cell-cell junctions [15]. This indicates that the site of RhoA activation, and which of its targets it stimulates, determines whether it inhibits or stimulates junction disruption.

### 3.2 Signals that reduce permeability

Stimuli that reduce permeability and promote endothelial barrier function generally act through three small GTPases, Rap1, Rac1 and Cdc42, and as described above in some cases RhoA, which act together through inter-connected signaling networks (Fig. 2). For example, thrombin initially increases endothelial permeability through RhoA, but also increases S1P generation, which then stimulates Rac1 activation resulting in enhanced barrier integrity to reverse the thrombin-induced permeability [54].

Endothelial junctional integrity is enhanced by stimuli that elevate cAMP levels, such as adrenomedullin, prostacyclin, prostaglandin E2, and β-adrenergic agonists, which reduce EC permeability [55]. cAMP directly activates the RapGEF Epac, which activates Rap1 [56]. There are two Rap1 proteins, Rap1a and Rap1b. Rap1b is most highly expressed in ECs, but Rap1a depletion reduces EC barrier function more than Rap1b depletion, which could be explained by its colocalization with VE-cadherin at AJs [57].

Rap1 acts through multiple pathways to promote VE-cadherin-mediated adhesion and maintain barrier function [56]. First, it leads to activation of Rac1 and Cdc42, which in turn strengthen endothelial cell-cell junctions. For example, Epac1/Rap1 act via the Rac GEFs Tiam1 and Vav2 to promote Rac1 activation [55]. In addition, circulating erythrocytes or platelets release S1P that activates Rac1 downstream of Rap1 via Akt, leading to endothelial barrier stabilization [58], [59]. Rap1 also promotes the assembly of a junctional mechanosensing complex of PECAM1, VE-cadherin and VEGFR2 in response to shear stress, which then activates Rac1 via Vav2 and Tiam1 to increase barrier function [60], [61], [62]. Finally, Rap1 activates Cdc42 via the RhoGEF FGD5 to promote cell-cell junction stabilization [63], [64].

In addition to activating Rac1 and Cdc42, Rap1 acts via several mechanisms to reduce RhoA/ROCK activity resulting in increased endothelial barrier function (Fig. 2). It acts via its effector Rasip1, which recruits the RhoGAP ARHGAP29 to inhibit RhoA and ROCK activity [65], [66]. Rasip1 also decreases stress fiber formation and endothelial permeability by direct interaction with the transmembrane receptor heart of glass (HEG1) [67]. Furthermore, Rap1 controls the endothelial barrier by recruiting its effector CCM1/KRIT1 to EC junctions, which reduces stress fibers and RhoA activity in ECs [68], [69]. In contrast to Rap1, Rap2 depletion increases endothelial barrier resistance, although the mechanism whereby Rap2 alters barrier function is not known [70].

Rac1 increases EC junction stability and hence reduces permeability, both by stimulating extension of lamellipodia to close intercellular gaps and by inducing assembly of cortical F-actin bundles and reducing actomyosin tension [71], [72]. In addition, shear stress acts via VE-cadherin, Tiam1 and Rac1 to reduce the level of tyrosine phosphorylation on occludin, leading to barrier enhancement [73], although the tyrosine phosphatase involved in this pathway has not been identified, Angiopoietin-1 reduces occludin tyrosine phosphorylation via the protein tyrosine phosphatase N-2 and promotes occludin interaction with ZO-1 to enhance TJs [74], and thus it will be interesting to test if this phosphatase acts downstream of Rac1.

Rac1 acts through several downstream effectors to mediate junction stabilization, including IQGAP1, which binds to Rac1 and Cdc42, and interacts with activators of actin polymerization (N-WASP, Arp2/3 complex) to promote AJ assembly [75], [76]. By contrast, Rac1 has been reported to increase permeability downstream of VEGF via PAK-mediated phosphorylation of a highly conserved motif within the intracellular tail of VE-cadherin, Ser665, resulting in VE-cadherin internalization [47]. VEGF activates VEGFR2 which associates with VE-cadherin and activates Rac1 via Src and the RacGEF Vav2 [47]. VEGF can also act via the phosphatidylinositol (3,4,5)-trisphosphate-dependent Rac exchanger 1 (P-Rex1) to activate Rac1 and increase permeability [77]. It is therefore likely that the effect of Rac1 on permeability depends on the cellular context.

As well as being activated downstream of Rap1, Rac1 is locally activated at cell-cell junctions, in part via the RhoGEFs Trio and Tiam1, which interact with VE-cadherin [44], [78], [79], [80]. On the other hand, inflammatory mediators such as LPS, TNFα, angiotensin 2 and thrombin reduce Rac1 activity resulting in junction opening and increased permeability [81]. RhoB inhibits Rac1 activity at junctions by reducing trafficking of Rac1 to cell-cell junctions. RhoB expression is induced by the inflammatory cytokines TNFα and IL-1β, and promotes sustained EC contraction upon thrombin exposure in the context of inflammation [46].

Like Rac1, Cdc42 is important for maintaining AJs [82], [83], [84]. For example, Cdc42 restores endothelial barrier function upon thrombin stimulation [85]. Increased endothelial permeability induced by loss or destabilization of VE-cadherin increases Cdc42 activity [84], [86], which presumably promotes junctional restoration. In vivo, Cdc42 is required for endothelial cell-cell adhesion during development, and is necessary for endothelial polarity [87].

Cdc42 acts via several mechanisms to stimulate AJ assembly. It promotes the post-Golgi transport of VE-cadherin to AJs [88]and controls α-catenin binding with β-catenin and interaction of the VE-cadherin complex with the actin cytoskeleton [84]. In addition, Cdc42 controls actin organization thought two pathways: first via its effectors PAK2 and PAK4 that regulate junctional actomyosin, and second via N-WASP that is required for junctional actin assembly [87]. Cdc42 has also been reported to act through the atypical protein kinase C PKCι to mediate endothelial cell-cell adhesion in vivo [89].

Overall, endothelial permeability reflects the combined actions of multiple signaling molecules, including small GTPases, kinases and phosphatases, which directly modify junctional molecules as well as act on the associated actin cytoskeleton to alter junctional integrity (Fig. 2).

## 4 Signaling in endothelial junctions and leukocyte transendothelial migration

Leukocyte TEM is essential both in immunosurveillance and during tissue damage or infection [90], [91]. TEM is a unique process in which a leukocyte squeezes itself through the endothelium, and involves dynamic signaling between leukocytes and ECs leading to the changes in morphology required for TEM (Fig. 3).

Leukocytes cross the endothelium via a multistep cascade involving leukocyte capture, rolling, adhesion, and crawling on ECs followed by leukocyte migration across the endothelium in the presence of shear forces exerted by blood flow (Fig. 3) [92], [93], [94]. Leukocytes can cross the endothelium through two different routes: paracellular, through the cell-cell junctions, or transcellular, throught the endothelial cell body [95], [96]. The choice of route depends on the transmigrating cell type, the site of TEM and the type of vascular endothelium [97], [98]. Furthermore, the route of diapedesis depends on endothelial junctional integrity: T-cells are more likely to take a transcellular route when junctions are strengthened [99].

Several adhesion molecules expressed by ECs contribute to TEM, including ICAM-1 and/or VCAM-1 on the luminal surface and PECAM-1 and/or JAMs on the basolateral membranes [8], [93], [100]. The expression and regulation of these molecules depends on the shear stress of the blood flow, proinflammatory cytokines and chemokines, the vascular bed and the stiffness of the tissue [101], [102].

### 4.1 Leukocyte crawling and endothelial extension of docking structures

Leukocytes initially crawl on ECs before arresting and transmigrating. The leukocyte integrin LFA-1/αLβ2 interaction with ICAM-1 activates RhoA [94]. RhoA promotes stiffening of the endothelial surface, enhancing leukocyte migration over it and hence the ability of leukocytes to find a site for TEM. ICAM-1 activates RhoA through the RhoGEF LARG (Leukemia-assiciated Rho GEF, ARHGEF12), and depletion of endothelial LARG reduces leukocyte crawling on ECs [103].

ECs often extend dynamic microvillus-like protrusions known as docking structures or transmigratory cups around adherent leukocytes (Fig. 3) [94]. These structures contribute to TEM, probably by increasing leukocyte-endothelium contact area with minimal barrier disruption and to help leukocytes extend protrusions to cross the endothelium [104]. Initially ICAM-1 and VCAM-1 cluster in ring-like structures around the leukocyte, followed by apical membrane protrusion. Anchorage of ICAM-1 to the actin cytoskeleton via adaptor proteins, such as filamin and cortactin, is essential for formation of ICAM-1 rings and subsequent leukocyte TEM [105], [106], [107].

Rac1 is required for ICAM-1 clustering, while RhoG controls membrane protrusion (Fig. 3). RhoG is activated by ICAM-1 binding to the RhoG-specific GEF SGEF [108]. ICAM-1 also associates with and activates the GEF Trio, which activates Rac1 and then RhoG via its two different RhoGEF domains [109]. Interestingly, Trio expression is strongly upregulated by TNFα stimulation, suggesting docking structure assembly is coupled to induction of ICAM-1 and VCAM-1 [109].

### 4.2 Crossing the endothelium and closing the transmigration pore

For paracellular TEM, two signaling events occur once the leukocyte is anchored to the endothelium: local VE-cadherin internalization via clathrin-coated vesicles and influx of membrane and membrane proteins including PECAM-1 from a lateral border recycling compartment (LBRC) to the TEM site [110], [111], [112], [113].

Junction opening involves VCAM-1 signaling to VE-cadherin. VCAM-1 interaction with the leukocyte integrin VLA-4 induces Rac1 activation and subsequent production of intracellular reactive oxygen species (ROS) by NADPH oxidase [114]. This in turn activates the proline-rich tyrosine kinase (Pyk2) [115] which then phosphorylates VE-cadherin on Tyr658 and Tyr731 resulting in local loss of VE-cadherin function, junction opening and increased TEM (Fig. 3) [36], [116], [117], [118]. Inhibition of this pathway, through the PI3-kinase p110α, Pyk2 and Rac1, reduces TEM [114], [118], [119], [120]. Rac1, ROS and Pyk2 also induce VE-PTP dissociation from VE-cadherin, leading to increased tyrosine phosphorylation of VE-cadherin and VE-cadherin internalization thereby facilitating transmigration [3], [31], [113], [121]. Furthermore, endothelial signaling stimulates junctional actomyosin contractility and acts on cell-cell junctional proteins, leading to localised and transient junctional disassembly essential for leukocyte TEM [94], [122].

Multiple proteins are associated with the LBRC fraction during TEM including PECAM-1, which is important for TEM [123], [124]. PECAM-1 homotypic interaction starts the recruitment of the LBRC to the site of leukocyte interaction. This is linked to localised recruitment of the Ca^2+^ channel TRPC6, which increases intracellular Ca^2+^ and is required for the final stage of TEM, similar to PECAM-1 [125]. Increased intracellular Ca^2+^ triggers actomyosin contractility by myosin light chain kinase (MLCK), which is required for TEM [102]. PECAM-1 is also involved in transcellular TEM, since ICAM-1 clustering on ECs is not sufficient to promote the transcellular pathway if PECAM-1 is blocked [126].

Endothelial RhoA is activated locally by ICAM-1 during TEM throught recruitment of the RhoGEFs LARG and Ect2 [45], [97]. RhoA activity is highest during the final stage of extravasation, and mediates endothelial F-actin remodelling to form ring structures around transmigrating leukocytes, which both prevent vascular leakage during leukocyte diapedesis and promote pore closure and transmigration [97].

## 5 Conclusions and future perspectives

Endothelial permeability and leukocyte TEM involve similar intracellular signaling mechanisms, including Rho and Rap GTPase signaling, cell-cell junction and F-actin remodelling, and thus it has been difficult to separate the two processes mechanistically. However, a key difference between them is when and where the signals occur in ECs. For leukocyte TEM, receptors are activated locally in ECs beneath and around the leukocyte [94], whereas for endothelial permeability the receptors are generally activated across the whole EC plasma membrane and/or cell-cell junctions [4]. In addition, TEM does not involve an increase in vascular permeability, rather that mechanisms are in place to ensure minimal leakage [36], [97], [127]. Moreover, stimuli leading to vascular permeability generally act within minutes [23], whereas leukocyte TEM during inflammation requires upregulation of leukocyte-binding receptors on ECs [91].

Although several signaling mechanisms are similar between endothelial permeability and TEM, there are also signals that differ, which could be manipulated to inhibit one or the other process. For example, differences in VE-cadherin tyrosine phosphorylation between the two processes have been reported [36], [128]. Future research should identify how these phosphorylation sites mediate local versus global changes to junctions in ECs. In addition, RhoG is so far only implicated in TEM [108]. On the other hand, Rap1, RhoA and Rac1 are involved in both processes. It is likely, however, that they are part of different protein complexes because the transmembrane receptors involved in permeability and TEM are different. Although several GEFs have been identified to contribute to permeability and TEM, future research should aim to identify which downstream targets and GAPs of Rho and Rap GTPases are critical to vascular permeability versus leukocyte TEM.

In contrast to leukocyte TEM, comparatively little is known about the contribution of EC signaling to cancer TEM. Multiple receptors on ECs have been implicated in mediating cancer cell TEM, but little is known of their mechanistic roles beyond simply facilitating adhesion [129]. Recently, Ephrin-1 was identified as a ligand on ECs that stimulates tyrosine phosphorylation of EphA2 on cancer cells, inducing cell-cell repulsion and leading to decreased TEM of breast cancer cells [130]. It will be interesting to determine how other receptors contribute to dynamic signaling between cancer cells and ECs.

In conclusion, changes to vascular permeability and leukocyte TEM are orchestrated by a combination of small GTPases, protein kinases and phosphatases, which coordinate changes to endothelial cell-cell junctions with the actin cytoskeleton. Targeting these signaling molecules could be used to reduce inflammation and auto-immune diseases.
